# Abnormal neonatal brain microstructure in gestational diabetes mellitus revealed by MRI texture analysis

**DOI:** 10.1038/s41598-023-43055-4

**Published:** 2023-09-21

**Authors:** Zhi Feng, Yurong Zheng, Ping Wang, Liang Xue, Mingling Yu, Zhitao Deng, Xiaoping Lei, Guangxiang Chen

**Affiliations:** 1 Department of Radiology, The Affiliated Hospital of Southwest Medical University, No.23 Tai Ping Street, Luzhou, 646000 Sichuan China; 2 Division of Neonatology, Department of Pediatrics, The Affiliated Hospital of Southwest Medical University, Luzhou, 646000 Sichuan China; 3 Department of Perinatology, The Affiliated Hospital of Southwest Medical University, Luzhou, 646000 Sichuan China; 4 Sichuan Clinical Research Center for Birth Defects, Luzhou, 646000 Sichuan China

**Keywords:** Diseases, Medical research, Neurology

## Abstract

To investigate the value of MRI texture analysis in evaluating the effect of gestational diabetes mellitus (GDM) on neonatal brain microstructure development, we retrospectively collected images of neonates undergoing head MRI scans, including a GDM group (N1 = 37) and a healthy control group (N2 = 34). MaZda texture analysis software was used to extract the texture features from different sequence images and perform dimensionality reduction, and then the texture features selected by the lowest misjudgement rate method were imported into SPSS software for statistical analysis. In our study, we found that GDM affects the development of the microstructure of the neonatal brain, and different combinations of texture features have different recognition performances, such as different sequences and different brain regions. As a consequence, texture analysis combining multiple conventional MRI sequences has a high recognition performance in revealing the abnormal development of the brain microstructure of neonates born of mothers with GDM.

## Introduction

Gestational diabetes mellitus (GDM) refers to any degree of glucose intolerance that develops or is first diagnosed in the mother during pregnancy^1,2^ and has recently been defined as "diabetes diagnosed in the second or third trimester of pregnancy without significant diabetes prior to pregnancy"^3^. GDM is the most common complication of pregnancy, with prevalence rates ranging from 1 to 20% and increasing yearly worldwide. GDM has been identified as one of the major barriers to improving maternal and child health^4,5^. Previous studies have found that 1 in 7 newborns is born to mothers with GDM^1,6,7^. Such offspring are at increased risk of multiple direct complications, including macrosomia, preterm birth, labor injury, shoulder dystocia, neonatal hypoglycemia, respiratory distress, hyperbilirubinemia and low Apgar scores^4,8–10^. In terms of long-term effects, GDM offspring are also at increased risk for diabetes and cardiovascular disease^7,8,11,14^.

Infant brain development is particularly sensitive to the health conditions of the mother. Maternal hyperglycemia leads to excessive production of proinflammatory cytokines in the placenta and directly affects the fetus, and neonatal hyperglycemia also promotes the production of interleukin 1β (IL-1β), tumor necrosis factor α (TNF-α) and toll-like receptor activity in spleen cells. The abnormal alterations lead to chronic low-grade systemic inflammation and induce the proinflammatory response of activated microglia to increase the sensitivity of inflammatory responses of the central nervous system and ultimately affect fetal and neonatal brain development^2^. Another possible mechanism linking GDM to abnormal brain development in neonates is iron deficiency caused by the rapid addition of red blood cells. Iron is involved in oxygen transport and organogenesis. Low iron promotes specific apoptosis and immature oligodendrocytes, resulting in hypomyelination and a profound influence on neural development^5^. In addition, learning and memory deficits are strongly associated with changes in insulin signaling within the brains of GDM offspring^12^.

To date, understanding of the effects of gestational diabetes on brain development has been largely based on an assessment of the growth and development of GDM offspring, the determination of some specific biological factors, genetic testing and animal experiments^2,10,12,13,15^. Few studies have used neuroimaging examinations to explore the effects of GDM on offspring brain development. One study using diffusion tensor imaging found that fasting glucose concentration during pregnancy in nonobese mothers was negatively correlated with the fractional anisotropy (FA) in the right amygdala of newborns^16^. Another neuroimaging study on GDM offspring showed that the FA of the corporis callosi splenium, posterior limb of the internal capsule, and thalamus of the GDM group were significantly lower than those of the control group, suggesting that the offspring of GDM may present abnormal white matter microstructure related to hypomyelination^5^. A study using an animal model reported that maternal hyperglycemia tended to cause fetal hyperglycemia and iron deficiency, affecting the neural development of several fetal brain regions, such as the striatum and hippocampus^12^. Magnetic resonance imaging (MRI) is widely used to acquire images and diagnose diseases in clinical practice and can provide multiparameter images such as T1-weighted imaging (T1WI), T2-weighted imaging (T2WI), fluid-attenuated inversion recovery (FLAIR) and diffusion-weighted imaging (DWI)^17^. Currently, MRI is considered an effective tool for accurately evaluating changes in the neonatal brain and the relationship between these changes and cognitive outcomes. However, previous studies mainly used conventional MRI or diffusion imaging but did not further mine the information contained in the imaging data of GDM offspring. Furthermore, neurological dysfunction has also been reported to occur in some neonates with normal conventional MRI findings^18,19^. Texture analysis, an image pattern extraction technique based on microstructural information representation, can extract data such as gray level intensity, pixel position, and the arrangement and relationship between voxel intensity from medical images, allowing quantification of the gray levels inside an image by measuring the spatial relationships through different quantitative values^20–22^. It is a well-known medical image processing approach widely used in neonatal acute bilirubin encephalopathy, perinatal asphyxia neonatal brain injury, Parkinson's disease and other brain diseases^23–25^.

To our knowledge, no studies have used MRI texture analysis to explore neural development in newborns of mothers with GDM. Therefore, our main study objective was to investigate the value of MRI image texture analysis in assessing the effect of GDM on the development of the neonatal brain microstructure and to identify the most valuable texture features with the best diagnostic performance, providing objective evidence for formulating early intervention strategies to reduce or avoid the occurrence of neurodevelopmental abnormalities in infants exposed to GDM.

## Results

### Demographic and clinical features

A total of 71 participants were enrolled, including 37 neonates in the GDM group (21 males and 16 females) and 34 neonates in the healthy control group (23 males and 11 females). The demographic and clinical details are listed in Table 1. There were no significant differences in sex (*p* = 0.35), gestational age (*p* = 0.93), days since birth (*p* = 0.92), body length (*p* = 0.48), weight (*p* = 0.45), head circumference (*p* = 0.99), neonatal blood glucose (*p* = 0.60), blood pH value (*p* = 0.22) or Apgar score (*p* = 0.53) between the two groups.

**Table 1:** General data of the two groups.

	GDM group		Control group		t/z/*χ*^2^	*P* value
	$\overline{X}$/$M$	$S$/$IQR$	$\overline{X}$/$M$	$S$/$IQR$		
Male/female	21/16		23/11		0.89	0.35
Gestational age (weeks)	35.57	34.43–37.93	35.50	34.54–38.21	− 0.09	0.93
Days since birth	7	5–9.50	7	5–9	− 0.10	0.92
Body length (cm)	45.95	2.90	45.41	3.36	0.72	0.48
Weight (g)	2450	2120–2880	2450	2010–2772.5	− 0.76	0.45
Head girth (cm)	32.09	1.35	32.09	1.90	0.07	0.99
Neonatal blood glucose value (mmol/l)	2.90	2–4	2.85	2.4–4.03	− 0.52	0.60
Blood PH	7.30	0.07	7.33	0.07	0.73	0.22
Apgar score (1 min)	9	8–10	10	7.75–10	− 0.64	0.53
Apgar score (5 min)	10	9–10	10	9–10	− 0.22	0.83
Apgar score (10 min)	10	10	10	10	− 0.05	0.96

### Texture feature dimension reduction and regression analysis

The results show that the combined MI + PA + F dimension reduction method and LDA method yielded the lowest misjudgment rate of 18.80%. Every ROI in a single sequence captured 30 texture features using the MI + PA + F dimension reduction method, and 330 texture features were obtained from the selected 11 ROIs in each sequence. A total of 1320 texture features from T1WI, T2WI, FLAIR and DWI sequences were imported into SPSS software. There were no significant differences in the texture features in the right caudate nucleus, right lenticular nucleus and right thalamus (*P* > 0.05), and the 203 texture features from the other 8 brain regions showed significant differences (*P* < 0.05) between the two groups. Finally, the 203 features with statistical differences were input into the binary logistic regression model, and the results showed that 29 features had a better fitting effect through the Hosmer‒Lemeshow test; that is, the prediction effect of each texture feature was better (*P* < 0.05).

### ROC curve analysis

**Figure 1:**
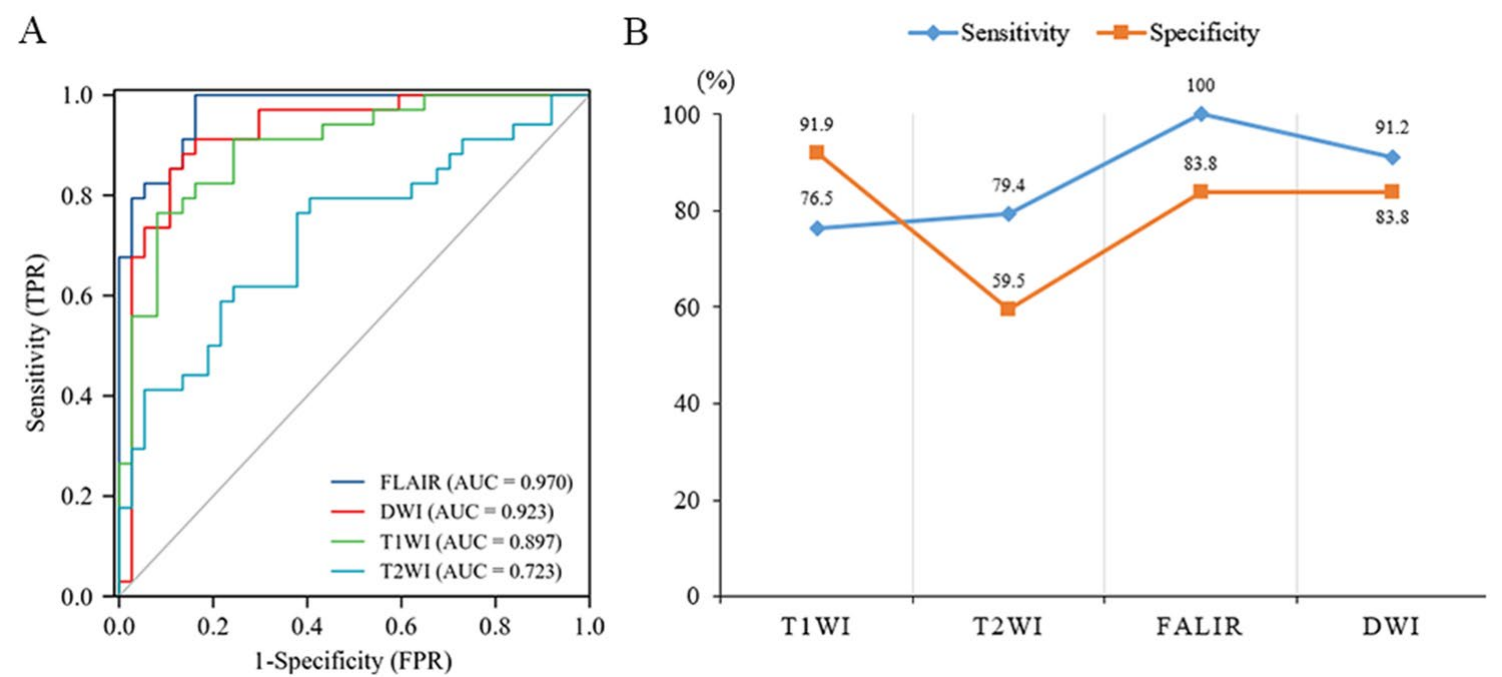
(**A**) The recognition performance of texture features from individual sequences. In order of decreasing recognition performance, the sequences were FLAIR, DWI, T1WI, and T2WI (AUC = 0.970, 0.923, 0.897, and 0.723, respectively). (**B**) Linear graphs of sensitivity and specificity for different sequences.

The recognition performances of the texture features from different sequences were different, as shown in Table 2. The texture features obtained from the FLAIR sequence had the highest AUC (0.970), followed by those from the DWI, T1WI and T2WI sequences. In addition, the texture features of the FLAIR sequence also had the highest sensitivity, and the texture features of the T1WI sequence had the highest specificity compared with those from the other sequences (Fig. 1A,B).

**Table 2:** Combined recognition performance of texture features from different brain regions for each sequence.

Sequence	AUC	95% confidence interval (CI)	Sensitivity (%)	Specificity (%)
T2WI	0.723	0.602–0.843	79.4	59.5
T1WI	0.897	0.825–0.970	76.5	91.9
FLAIR	0.970	0.939–1.000	100.0	83.8
DWI	0.923	0.856–0.990	91.2	83.8

**Figure 2:**
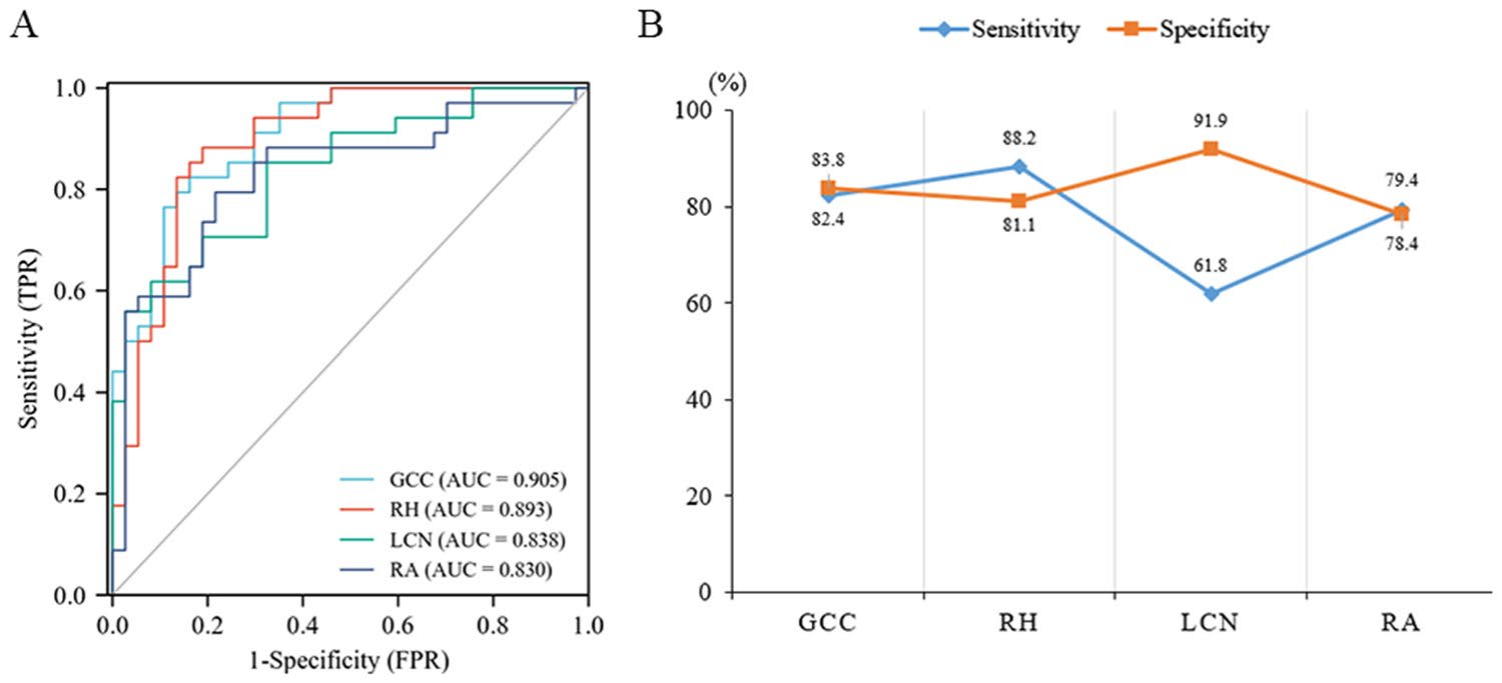
(**A**) The recognition performance of textural features in individual brain regions. (**B**) Linear graphs of sensitivity and specificity in different brain regions.

The recognition performances of texture features in different brain regions also differed, as shown in Table 3. The genu of the corpus callosum, right hippocampus, left caudate nucleus and right amygdala had high AUC values of 0.905, 0.893, 0.838 and 0.830, respectively (Fig. 2A,B).

**Table 3:** Combined recognition performance of texture features from the different sequences for each brain region.

Encephalic region	AUC	95% CI	Sensitivity (%)	Specificity (%)
Genu of the corpus callosum (GCC)	0.905	0.840–0.971	82.4	83.8
Left hippocampus (LH)	0.757	0.643–0.870	67.6	78.4
Right hippocampus (RH)	0.893	0.817–0.968	88.2	81.1
Left caudate nucleus (LCN)	0.838	0.746–0.930	61.8	91.9
Left amygdala (LA)	0.746	0.630–0.861	82.4	64.9
Right amygdala (RA)	0.830	0.729–0.930	79.4	78.4
Left thalamus (LT)	0.719	0.599–0.838	70.6	67.6
Left lenticular nucleus (LLN)	0.776	0.667–0.885	91.2	56.8

**Figure 3:**
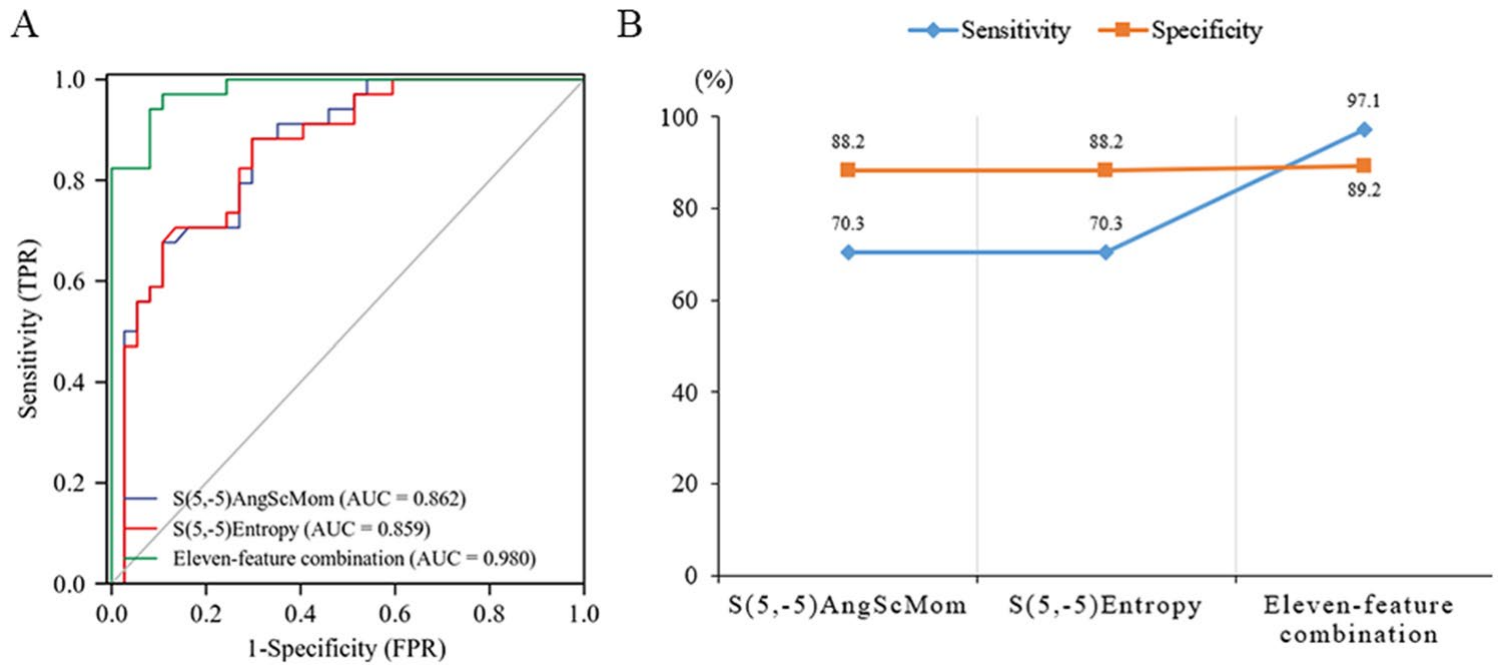
(**A**) The recognition performance of S(5, 5) AngScMom and S(5, 5) Entropy of the genu of the corpus callosum from the FLAIR sequence and the combined recognition performance of the 11 texture features with AUC > 0.7. (**B**) Linear graphs of their sensitivity and specificity.

In single texture feature analysis, the two texture features (S(5, 5)AngScMom and S(5, 5)Entropy) in the genu of the corpus callosum from the FLAIR sequence obtained high AUC values (0.862 and 0.859, respectively). Among the 29 texture features obtained from the T1WI, T2WI, FLAIR and DWI sequences, 11 texture features, mainly from the FLAIR and DWI sequences, had AUCs greater than 0.7, as shown in Table 4. Combining the 11 texture features, the AUC value, sensitivity and specificity for identifying GDM neonates were 0.98, 0.971 and 0.892, respectively (Fig. 3A,B).

**Table 4:** Eleven texture features with the best recognition performance in all selected brain regions of all sequences and their combined recognition performance.

Sequence	Encephalic region	Texture feature	AUC	95% CI	Sensitivity (%)	Specificity (%)
T2WI	RA	S(4,0)DifEntrp	0.723	0.602–0.843	79.4	59.5
FLAIR	GCC	S(5, -5)AngScMom	0.862	0.777–0.947	70.3	88.2
		S(5, -5)Entropy	0.859	0.772–0.946	70.3	88.2
	RH	WavEnLH_s-1	0.781	0.672–0.889	78.4	67.6
	LCN	S(5,0)Contrast	0.727	0.610–0.845	89.2	47.1
DWI	GCC	S(2,2)SumAverg	0.719	0.599–0.839	51.4	94.1
	LH	S(4,4)Entropy	0.709	0.585–0.833	81.1	55.9
	RH	S(2, -2)Entropy	0.742	0.627–0.858	85.3	59.5
		S(3,3)SumEntrp	0.723	0.602–0.844	61.8	78.4
		S(3, -3)Entropy	0.734	0.617–0.851	52.9	83.8
		S(4, -4)DifEntrp	0.763	0.652–0.873	64.7	78.4
Eleven-feature combination			0.980	0.957–1.000	97.1	89.2

## Discussion

In this study, texture analysis of MR images became an effective tool for the quantitative identification of differences in brain microstructure development between GDM neonates and healthy controls. By extracting and analyzing the texture features of the studied brain regions from T1WI, T2WI, FLAIR and DWI sequences, we obtained 29 texture features with AUC values greater than 0.6. By synthesizing the statistically significant texture features extracted from each sequence, we found that the recognition performance of those from the different sequences was different. The results of this study indicated that the AUC value and sensitivity of the FLAIR sequence were the highest at 0.97 and 100.0%, respectively. Taken together, we concluded from these findings that the FLAIR sequence had the best recognition performance. It should be emphasized that the sequence with the highest specificity was T1WI, with a value of 91.9%. In addition, the results showed that the number of texture features with recognizable value in the T2WI sequence was the least, which may indicate that the value of the T2WI sequence in detecting brain microstructure abnormalities in offspring of GDM was relatively low.

We identified some abnormal development of the deep gray matter and white matter microstructure in GDM neonates. The recognition performance of the texture features in different brain regions was also different; specifically, the genu of the corpus callosum, the right hippocampus, the left caudate nucleus and the right amygdala had the best combined recognition performance. Previous studies using the DTI sequence^5,12,16^ found microstructural abnormalities of the thalamus and right amygdala, which are consistent with the results of this study. Xuan et al*.* found that the abnormal microstructure of the splenium of the corpus callosum in GDM neonates^5^, together with the abnormal microstructure of the genu of the corpus callosum revealed by our study, is conducive to further demonstrating and integrating the influence of GDM on the neonatal corpus callosum microstructure. Combined with the results of animal experiments conducted by De et al.^12^ and the abnormal development of the hippocampus in the GDM group in this study, it is preliminarily concluded that the influence of GDM on the brain development of its offspring may last from the fetal to neonatal period. Although microstructural abnormalities in the genu of the corpus callosum, left caudate nucleus and left lenticular nucleus of GDM neonates were not mentioned in previous neuroimaging studies, our study used texture analysis to quantitatively detect subtle microstructural abnormalities in the above brain regions. These differences may be due to the different sensitivities of the imaging methods for subtle pathological alterations. Therefore, more research data are needed to further explore and confirm the above results.

Among the 29 texture features that were finally obtained, we found a total of 11 texture features with AUCs greater than 0.7. Combining the above 11 texture features was helpful in identifying the effects of GDM on the development of the neonatal brain microstructure. Notably, only the AUC values of the S(5, 5)AngScMom and S(5, 5)Entropy features in the genu of the corpus callosum in the FLAIR sequence are both greater than 0.8, indicating that these two texture features from a single brain region in a single sequence had good recognition performance. AngScMom reflects the uniformity of the gray distribution and texture thickness of the image, and entropy represents the degree of uncertainty (a measure of randomness). Both of these features are derived from the coocurrence matrix and reflect the spatial correlation characteristics of grayscale on the image^18,25,26^. The results may be associated with the disrupted microstructural features of the neonatal brain, especially the genu of the corpus callosum in the GDM group. The potential pathophysiological mechanism of the findings is that neuroinflammation caused by GDM leads to abnormal neuron distribution and apoptosis in the neonatal brain^2,16^. In addition, iron deficiency in newborns of diabetic mothers promotes neuronal apoptosis and immature oligodendrocytes, resulting in hypomyelination and abnormal cortical connections^5,12^. Overall, these results suggest that the texture features based on conventional brain images of neonates, especially the texture features of the genu of the corpus callosum in FLAIR sequence images, are of great value in revealing the effects of GDM on neonatal brain development.

The limitations of this study should be considered. First, the acquisition of brain images of neonates was relatively difficult, so the number of data samples was relatively small, and the possibility of occasionality in some results cannot be completely ruled out. Furthermore, because of the small sample size, subgroup analyses were not performed to exclude the effects of confounding factors such as the severity of GDM, duration of disease, and treatment scheme on the results. However, this study is the first to explore the neurodevelopmental abnormalities of GDM offspring by using MRI texture analysis, so a large amount of data is needed to further verify the results. Second, there may be some differences in the selected image levels and manual ROI segmentation in the study samples, and the extracted texture features of corresponding brain regions may not completely reflect the heterogeneity of the lesions. Thus, it is necessary to further verify the repeatability of the method in future studies. Third, this was a retrospective cross-sectional study, so some valuable clinical information was absent in the medical records, and the neurodevelopmental evolutional pattern of GDM offspring could not be explored. Prospective longitudinal studies investigating the long-term effects of GDM on neurodevelopment as well as relevant cognition and behavior in GDM offspring can be considered a future research direction.

In conclusion, our study used quantitative texture analysis based on conventional MRI to demonstrate that GDM may affect the development of the neonatal brain microstructure. Furthermore, this approach has a high recognition performance in detecting the abnormal development of neonatal brain microstructure before visible morphological abnormalities appear, especially the texture features of the genu of the corpus callosum from the FLAIR sequence, which have good recognition performance and may be a potential biomarker for the early identification and monitoring of GDM-related brain abnormalities in neonates and provide objective evidence for intervention strategies. In addition, our study only required conventional brain MRI of neonates and avoided the increase in scanning time for acquiring additional sequences because of the difficulty of cooperation for neonates during scanning, conducing the possibility for clinical practice.

## Materials and methods

### Participants

Conventional plain head MR scan images of 71 neonates in the Affiliated Hospital of Southwest Medical University were retrospectively collected, including 37 patients in the GDM group and 34 patients in the healthy control group. Clinical data mainly included gestational age, days since birth, body length, weight, head circumference, neonatal blood glucose, blood pH and Apgar scores (1–5–10 min).

The inclusion criteria of the GDM group were as follows: (1) the mother of the neonate was diagnosed with GDM (the diagnostic criteria for GDM according to the International Association of Diabetes and Pregnancy Study Groups Recommendations^27^); (2) in addition to GDM, the mother of the neonate had no other diseases affecting fetal development during pregnancy and delivery, such as severe anemia, serious lung disease, hypertension, etc.; and (3) neonatal Apgar score (1–5–10 min) ≥ 7 after birth, and clinical data were complete. The inclusion criteria of the healthy control group were as follows: (1) the mother was healthy, and the neonate did not suffer from any diseases affecting brain development or developmental malformations; and (2) neonatal Apgar score (1–5–10 min) ≥ 7 after birth. The exclusion criteria were as follows: (1) any diseases or developmental malformations that may affect brain development, such as respiratory distress, bilirubin encephalopathy, and infection; (2) a neonatal Apgar score (1–5–10 min) < 7; and (3) a history of improper postpartum care.

This study was in accordance with the World Medical Association Declaration of Helsinki and approved by the Ethics Committee of the Affiliated Hospital of Southwest Medical University. Confirms that informed consent was obtained from the parents or legal guardians of all participants.

### Image acquisition

Images were acquired from all participants within 2 weeks of birth. Axial scanning was performed on a Siemens Prisma 3.0 T MRI device with a 32-channel head phased-array coil and the following imaging parameters: ① T1-weighted imaging (T1WI): turbo spin echo (TSE), repetition time/echo time (TR/TE) 1600/8.6 ms, slice thickness 4 mm, slice gap 0 mm, matrix 256 × 230, field of view (FOV) 200 mmx200 mm; ② T2-weighted imaging (T2WI): TSE, TR/TE 5140/120 ms, slice thickness 4 mm, slice gap 0 mm, matrix 320 × 210, FOV 170 mmx180 mm; ③ T2 blade fluid-attenuated inversion recovery (FLAIR) imaging: TSE/inversion recovery, TR/TE 6000/123 ms, TI 1966 ms, slice thickness 4 mm, slice gap 0 mm, matrix 256 × 256, FOV 180 mmx180 mm; ④ Diffusion-weighted imaging (DWI): TSE, TR/TE 4000/64 ms, slice thickness 4 mm, slice gap 0 mm, matrix 128 × 128, FOV 180 mmx180 mm, b = 1000 s/mm^2^. Twenty brain images of each sequence of every subject were acquired.

### Image processing

#### Region of interest (ROI) selection

**Figure 4:**
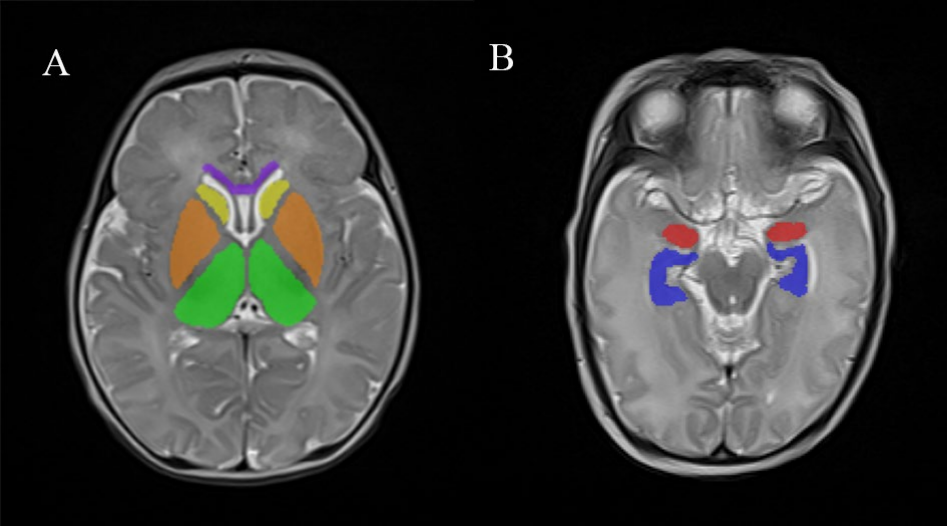
Example of a delineation of the investigated cerebral structures on T2W images. The different brain regions are represented by different colors. Purple represents the genu of the corpus callosum, yellow represents the caudate nucleus, orange represents the lenticular nuclei, green represents the thalamus, red represents the amygdala and blue represents the hippocampus.

Image quality was assessed to exclude motion artifacts, excessive noise and other poor image quality features. All images were exported from the hospital picture archiving and communication system (PACS) workstation in DICOM format, and the two slices with the best visualization of the studied brain regions in the axial view of each sequence were selected. The obtained images were imported into MaZda texture analysis software (Version 4.7, an open-source texture analysis software developed by Rhodes Industrial University, Poland, accessible at http://www.eletel.p.lodz.pl/mazda/). Using the principle of blinding, two radiologists mapped the ROIs of each patient. When there was disagreement about the delineation of the ROI, the ROI was adjusted after discussion between the two doctors and then verified by a senior radiologist. Finally, consistent results were obtained. We considered and investigated the alterations of texture features in the following structures: the hippocampus, amygdala, caudate nucleus, lenticular nucleus, thalamus and genu of the corpus callosum (left and right sides, for a total of 11 ROIs). All selected brain regions are related to GDM offspring or cognitive function according to previous studies^5,16,24,28–30^. All ROIs were manually delineated on the T2W images (Fig. 4) and mapped to the corresponding brain regions on the T1WI, FLAIR, and DWI.

#### Texture feature extraction

This process of texture feature extraction included the following steps: image import, ROI sketch and image normalization, ROI analysis and feature report acquisition. First, we imported a whole image selected into MaZda texture analysis software every time; second, the ROIs on the image were manually sketched and then set and saved in the image normalization mode to μ ± 3 SD (where μ is the average pixel gray level and SD is the standard deviation); finally, the sketched and normalized ROIs on the image were automatically analyzed, and the texture feature extraction reports of the ROIs were obtained by the software. The process of remaining images was successively repeated according to the above steps for feature extraction. The texture features extracted by MaZda software were classified into the following six categories: run-length matrix (RLM), coocurrence matrix (COM), absolute gradient (GRA), histogram, autoregressive model (AR) and wavelet transform (WAV)^31^. Each ROI can extract 300 texture features from a single sequence. A complete list of the individual texture features that MaZda can calculate, along with a short description of each feature, can be accessed at http://www.eletel.p.lodz.pl/programy/mazda/download/FeaturerList.pdf.

#### Texture feature selection

After texture feature extraction, we imported all texture feature extraction reports into MaZda software and performed dimensionality reduction. Four feature reduction methods, namely, the Fisher coefficient method (Fisher), minimizing classification error probability and average correlation coefficient method (POE + ACC), mutual information coefficient method (MI), and the combination of the above three methods (MI + PA + F), will obtain 10, 10, 10, and 30 optimal texture features, respectively. The B11 program analysis module embedded in MaZda software is used to analyze the optimal texture features obtained by the above four feature reduction methods. The analysis methods included principal component analysis (PCA), linear discriminant analysis (LDA), nonlinear discriminant analysis (NDA), and raw data analysis (RDA), and the analysis results were expressed as the false error rate (number of false errors/total number of patients). The feature reduction method with the lowest error rate can screen out the features with higher distinguishing ability so that enough relevant information for texture discrimination can be obtained when the number of features is limited^31^. Thus, the feature reduction method corresponding to the lowest error rate is selected as the optimal dimensionality reduction method in the present study.

### Statistical analysis

SPSS 22.0 statistical analysis software was used. The normality of the distribution of measurement data was assessed with a normality test. Data conforming to a normal distribution are expressed as the mean ± standard deviation $\left( {\overline{X} \pm S} \right)$ and as the median $\left( M \right)$ and interquartile range $\left( {IQR} \right)$ otherwise. Enumeration data are expressed as the number of patients or rate. The *χ*^2^ test was used for comparing the distribution of sex. The two independent-sample t-test (normal distribution) or the Mann‒Whitney *U* test (nonnormal distribution) was used to compare the differences in each measurement variable between the two groups of subjects, and *P* < 0.05 was considered statistically significant. A binary logistic regression model was used to analyze significantly different texture features between the groups, and the goodness of fit of the model was tested by the Hosmer‒Lemeshow method. The characteristic parameters finally included in the regression model were successively modeled according to different sequences, brain regions and combinations of the indexes. The receiver operating characteristic (ROC) curve was used to assess the performance of the model, and the area under the curve (AUC), sensitivity and specificity were obtained.

## Data Availability

Data relevant to the study are included in the article. In addition, the datasets generated during and/or analyzed during the current study are available from the corresponding author on reasonable request.
